# Three distinct mechanisms underlying human γδ T cell-mediated cytotoxicity against malignant pleural mesothelioma

**DOI:** 10.3389/fimmu.2023.1058838

**Published:** 2023-03-17

**Authors:** Yasuhiro Umeyama, Hirokazu Taniguchi, Hiroshi Gyotoku, Hiroaki Senju, Hiromi Tomono, Shinnosuke Takemoto, Hiroyuki Yamaguchi, Mohammed S. O. Tagod, Masashi Iwasaki, Yoshimasa Tanaka, Hiroshi Mukae

**Affiliations:** ^1^ Department of Respiratory Medicine, Graduate School of Biomedical Sciences, Nagasaki University, Nagasaki, Japan; ^2^ Center for Medical Innovation, Nagasaki University, Nagasaki, Japan; ^3^ Department of Respiratory Medicine, Senju Hospital, Sasebo, Japan; ^4^ Clinical Oncology Center, Nagasaki University Hospital, Nagasaki, Japan; ^5^ Center for Innovation in Immunoregulative Technology and Therapeutics, Graduate School of Medicine, Kyoto University, Kyoto, Japan

**Keywords:** γδ T cell, malignant pleural mesothelioma, natural killer receptor, nitrogen-containing bisphosphonate prodrug, phosphoantigen, immunotherapy

## Abstract

**Introduction:**

Malignant pleural mesothelioma (MPM) is a rare and highly aggressive thoracic tumor with poor prognosis and limited therapeutic options. Although immune checkpoint inhibitors exhibit a promising effect in some patients with unresectable MPM in clinical trials, the majority of MPM patients show only modest response rates to the currently available treatments. It is thus imperative to develop novel and innovative therapeutic modalities for MPM, including immune effector cell-based therapies.

**Methods:**

γδ T cells were expanded using tetrakis-pivaloyloxymethyl 2-(thiazole-2-ylamino) ethylidene-1,1-bisphosphonate (PTA) and interleukin-2, and the therapeutic potential of γδ T cells was examined through analyzing cell surface markers and cellular cytotoxicity against MPM in vitro using a europium chelate-based time-resolved fluorescence assay system and a luciferase-based luminescence assay system.

**Results and discussion:**

We successfully expanded γδ T cells from peripheral blood mononuclear cells of healthy donors and MPM patients. γδ T cells expressed natural killer receptors such as NKG2D and DNAM-1 and exhibited a moderate level of cytotoxicity to MPM cells in the absence of antigens. The inclusion of PTA, (*E*)-4-hydroxy-3- methylbut-2-enyl diphosphate (HMBPP) or zoledronic acid (ZOL) induced a TCR-dependent cytotoxicity in γδ T cells and secreted interferon-γ (IFN-γ). In addition, γδ T cells expressing CD16 exhibited a significant level of cytotoxicity against MPM cells in the presence of an anti-epidermal growth factor receptor (EGFR) mAb, at lower concentrations than in clinical settings, whereas a detectable level of IFN-γ was not produced. Taken together, γδ T cells showed cytotoxic activity against MPM in three distinct mechanisms through NK receptors, TCRs and CD16. Since major histocompatibility complex (MHC) molecules are not involved in the recognition, both autologous and allogeneic γδ T cells could be used for the development of γδ T cell-based adoptive immunotherapy for MPM.

## Introduction

Cancer is a major public health problem and the leading cause of deaths in developed countries. Malignant pleural mesothelioma (MPM) is a rare, but life-threatening malignancy that develops from pleural mesothelial cells lining around the lungs, and is typically caused by previous exposure to asbestos (1). Because a large amount of asbestos had been used in many industries such as shipbuilding and construction in 1970s - 1980s and MPM develops after long latent period (up to 30-50 years), the incidence of MPM appears to have only recently peaked. Whereas treatments with a combination of multi-modality therapies including surgery, radiation, and chemotherapy are recommended, most MPM patients are diagnosed as unresectable stage and receive conventional systemic chemotherapy (2). Therapeutic options of chemotherapy are thus very limited and the standard first-line chemotherapy for MPM has been pemetrexed and cisplatin or pemetrexed and carboplatin in this decade, resulting in poor prognosis (3–5).

Immunotherapy using immune checkpoint inhibitors (ICIs), has led to a revolution in the treatment of several solid tumors either as single agents or in combination. ICIs can promptly rejuvenate adaptive T cell immunity leading to durable antitumor responses in some cases with solid tumors (6). Recently, combined therapy with anti-programmed death 1 (PD-1) antibody nivolumab plus anti-cytotoxic T-lymphocyte 4 (CTLA-4) antibody ipilimumab significantly extended overall survival of MPM patients, compared to chemotherapy in patients with unresectable MPM in a clinical trial (7). Since the effect of ICIs combination therapy is still unsatisfactory including modest response rates and early acquired resistance, it is imperative to develop novel modalities for MPM, among which adoptive immunotherapy may have a potential to be a breakthrough as a novel therapeutic option for patients with MPM.

Adoptive transfer of T cells has attracted much attention as a new strategy of cancer immunotherapy. Using ex vivo-expanded T cells genetically-modified with a synthetic chimeric antigen receptor (CAR) derived from a monoclonal antibody, so-called CAR-T cell therapy, high rates of durable responses were observed in patients with B cell lymphoma (8). However, the effect of CAR-T cell therapy against solid tumors has been comparatively moderate (9), indicating that alternative strategies are required to develop more effective immunotherapy for solid tumors including MPM.

T cells express T cell receptors (TCRs), which are membrane-anchored heterodimeric proteins consisting of either α and β or γ and δ chains expressed as part of a complex with cluster of differentiation 3 (CD3). Most αβ T cells expressing α and β TCR chains recognize antigenic peptides restricted by the major histocompatibility complex (MHC) class I or II molecules with the aid of CD4 or CD8 co-receptors. By contrast, the majority of γδ T cells (Vγ9Vδ2-bearing γδ T cells, also termed Vγ2Vδ2) recognize isoprenyl diphosphates such as (*E*)-4-hydroxy-3-methylbut-2-enyl diphosphate (HMBPP) as a foreign antigen derived from pathogenic microbes and isopentenyl diphosphate (IPP) and dimethylallyl diphosphate (DMAPP) as self-antigens from a so-called mevalonate pathway in a butyrophilin (BTN) 3A1/2A1-dependent manner. It is worthy of note that treatment of cells with nitrogen-containing bisphosphonate (N-BP) drugs used for hypercalcemia of malignancy or osteoporosis, such as zoledronic acid, alendronate, ibandronate and pamidronate, inhibits farnesyl diphosphate synthase (FDPS), resulting in intracellular accumulation of IPP and DMAPP that stimulate γδ T cells. The role of Vγ9Vδ2-bearing γδ T cells in cancer immunity has not been fully clarified yet. We have previously developed a protocol to efficiently expand γδ T cells from peripheral blood to purities up to 95–99% with tetrakis-pivaloyloxymethyl 2-(thiazole-2-ylamino) ethylidene-1,1-bisphosphonate (PTA) and interleukin-2 (IL-2), and demonstrated that γδ T cells exhibited cytotoxicity against lung adenocarcinoma cells, strongly suggesting that γδ T cells might be utilized for adoptive immunotherapy for MPM (10).

In this study, we first expanded γδ T cells derived from healthy donors with PTA/IL-2 and examined the effect of them on MPM cells in the absence or presence of antigens or monoclonal antibodies. We then analyzed the phenotype and functions of γδ T cells derived from MPM patients and explored the possibility for the development of γδ T cell-based immunotherapy for MPM.

## Materials and methods

### Expansion of Vγ2Vδ2 T cells

Peripheral blood samples were collected from healthy volunteers and MPM patients after approval of the Institutional Review Board of Nagasaki University Hospital and with written informed consent. All procedures were performed in accordance with the Guidelines and Regulations of Nagasaki University Hospital. The blood sample (10 ml) was treated with 0.1 ml of heparin sodium solution (Mochida Pharmaceutical., Co., Ltd., Shinjuku-ku, Tokyo, Japan) and diluted with 10 ml of phosphate-buffered saline. The diluted blood (20 ml) was loaded onto Ficoll-Paque™ PLUS (20 ml, Cytiva, Shinjuku-ku, Tokyo, Japan) in a 50 ml conical tube (Corning Inc., Corning, NY), which was centrifuged at 600 x g at room temperature for 30 min without acceleration and deceleration. The lymphocyte layer was collected into a 50 ml conical tube and diluted with 35 ml of PBS. The mixture of peripheral blood mononuclear cells (PBMC) and Ficoll was centrifuged at 900 x g and 4 °C for 10 min. After the supernatant was removed, the resulting cell pellets were dispersed by tapping and resuspended in 13 ml of PBS. Then, the cell suspension was transferred into a 15 ml conical tube, which was centrifuged at 600 x g and 4 °C for 5 min. The supernatant was removed and the resulting cell pellets were dispersed by tapping, to which was added Yssel’s medium to give a cell concentration of 2 x 10^6^ cells/ml (11).

The PBMC suspension (1.5 ml) was placed in a well of a 24-well plate (Corning Inc.), to which was added 1.5 μl of 1 mM PTA stock solution (Techno Suzuta Co., Ltd., Heiwa-machi, Nagasaki, Japan) in dimethyl sulfoxide (Nacalai Tesque Inc., Nakagyo-ku, Kyoto, Japan) to give a final concentration of 1 μM. The plate was incubated at 37 °C with 5% CO_2_ overnight, to which was added interleukin-2 (IL-2, Shionogi Pharmaceutical Co., Ltd., Chuo-ku, Osaka, Japan) to give a concentration of 100 U/ml. After incubation at 37 °C with 5% CO_2_ for 24 h, the medium was replaced with Yssel’s medium containing 100 U/ml IL-2. On day 2 through day 5, the culture medium was supplemented with 100 U/ml of IL-2 and γδ cells were expanded. On day 6, 1.5 ml of Yssel’s medium was added to the well. After being mixed with a pipet, 1.5 ml of the cell suspension was transferred to another well. On day 7, the medium was replaced with the complete RPMI1640 medium plus 100 U/ml IL-2, Then, γδ T cells were incubated by day 11, placed at -80 °C, and then stored in liquid nitrogen until used. Cells were observed under a microscope (Nikon Corp., Minato-ku, Tokyo, Japan) on days 2 through 8, and the proportion of Vδ2-positive cells was determined by flow cytometry before and after expansion.

### Flow cytometric analysis

Cells (2 x 10^5^ cells) were dispensed into a 96-well round bottom plate (Corning Inc.) and stained with monoclonal antibodies (mAbs), including fluorescein isothiocyanate (FITC)-conjugated anti-TCR Vδ2 mAb (BD Biosciences, San Diego, CA) and phycoerythrin (PE)-conjugated anti-CD3 mAb (Tombo Biosciences, Co., Ltd., Kobe, Hyogo, Japan), anti-NKG2D, DNAM-1, or CD16 mAbs (BioLegend Japan, Bunkyo-ku, Tokyo, Japan) in 50 μl of PBS containing 2% fetal calf serum (FCS, Merck, Darmstadt, Hessen, Germany). After the plate was incubated on ice for 15 min, 200 μl of PBS/2% FCS was added to each well of the plate. Then, the plate was centrifuged at 600 x g and 4 °C for 2 min. After the supernatants were removed, the cell pellets were dispersed by vortexing and resuspended in 200 μl of PBS/2% FCS. The cells were washed two more times with 200 μl of PBS/2% FCS and resuspended in 400 μl of PBS/2% FCS. The cell suspensions were passed through a mesh filter and analyzed using a FACS Lyric flow cytometer (Becton Dickenson, Franklin Lakes, NJ). The cell population was visualized using FlowJo ver. 10 (FlowJo LLC, Ashland, OR). For analysis of the expression of epidermal growth factor receptor (EGFR), MPM cell lines were stained with biotinylated anti-human EGFR mAb, followed by anti-biotin protein-labeled green fluorescence protein (Techno Suzuta Co., Ltd.)

### Cytotoxicity assay by time-resolved fluorescence spectroscopy

Cytotoxic activity of γδ T cells against MPM cells was measured using a non-radioactive cellular cytotoxicity assay kit (Techno Suzuta Co., Ltd.). MESO-1 and MESO-4 human malignant pleural mesothelioma (MPM) cell lines were purchased from American Type Culture Collection (Manassas, VA). Daudi human Burkitt’s lymphoma, RPMI8226 human multiple myeloma, K562 human erythrocytoma, RAMOS-RAI human Burkitt’s lymphoma and U937 human monocyte-like cell lines were obtained from Health Science Research Resources Bank, Sennan, Osaka, Japan. The cells lines were maintained in complete RPMI1640 medium. Tumor cells (1 x 10^6^ cells in 1 ml) were pulsed with 25 μM bis(butyryloxymethyl) 4’-(hydroxymethyl)-2,2’:6’,2’’-terpyridine-6,6’’-dicarboxylate (BM-HT, Techno Suzuta Co., Ltd.) at 37 °C for 15 min. When BM-HT was internalized into tumor cells, the compound was hydrolyzed by intracellular esterases to yield 4’-(hydroxymethyl)-2,2’:6’,2’’-terpyridine-6,6’’-dicarboxylate (HT). After the cells were washed three times with 5 ml of complete RPMI140 medium, cell pellets were resuspended in 20 ml of the complete RPMI1640 medium. The tumor cell suspensions (5 x 10^3^ cells/100 μl) were dispensed into a 96-well round bottom plate, to which were added 100 μl of a serial dilution of γδ T cells at E/T ratios of 40:1, 20:1, 10:1 and 5:1 for hematopoietic tumor cells and those of 200:1, 100:1, 50:1 and 25:1 for MPM cells. The plate was centrifuged at 200 x g for 2 min and then incubated at 37 °C with 5% CO_2_ for 40 min. Detergent (Techno Suzuta Co., Ltd.) was added to wells at a final concentration of 5 x 10^-5^ M for maximum release and the plate was incubated at 37 °C for 20 more min. After the cell suspensions were mixed, the plate was centrifuged at 600 x g for 2 min and the supernatants (25 μl each) were transferred into a new 96-well round bottom plate containing 250 μl of europium (Eu^3+^) solution in 0.3 M sodium acetate buffer, pH 4 (Techno Suzuta Co., Ltd.). The culture supernatant/Eu mixtures (200 μl each) were transferred to a 96-well optical plate (Thermo Fisher Scientific Inc., Waltham, MA). Time-resolved fluorescence (TRF) was measured through an ARVO or NIVO multi-plate reader (PerkinElmer Inc., Waltham, MA). All measurements were performed in triplicate. Specific lysis (%) was calculated as 100 x [experimental release (counts) – spontaneous release (counts)]/[maximum release (counts) – spontaneous release (counts)]. To examine the effect of PTA on the cellular cytotoxicity, MESO-1 and MESO-4 were pulsed with 100 nM or 1 μM PTA at 37 °C for 1 h before being pulsed with BM-HT.

### Cytotoxicity assay by luminescence spectroscopy

For determination of NK-like activity of γδ T cells, MESO-1 and MESO-4 cell suspensions (2 x 10^4^ cells/200 μl) were placed in a 96-well flat bottom plate and incubated at 37 °C with 5% CO_2_ overnight. After the culture supernatants were aspirated, γδ T cells were added to the plate at effector-to-target (E/T) ratios of 0:1, 25:1, 50:1, 100:1 or 200:1 and incubated at 37 °C with 5% CO_2_ for 72 h. Then, the culture supernatants were aspirated and the wells were gently washed three times with 200 μl of complete RPMI1640 medium. To each well was added 100 μl of CellTiterGlo^®^ Reagent (PerkinElmer Inc.), and the cell lysates were transferred into a 96-well Optiplate (PerkinElmer Inc.). Luminescence was measured through an ARVO or NIVO multi-plate reader (PerkinElmer Inc.). All measurements were performed in triplicate.

For determination of cellular cytotoxicity of γδ T cells in the presence of PTA, HMBPP, (1-hydroxy-2-imidazol-1-yl-1-phosphonoethyl)phosphonic acid (zoledronic acid, ZOL), or anti-EGFR mAb, MPM cell suspensions (2 x 10^4^ cells/200 μl) were placed in a 96-well flat bottom plate and incubated at 37°C with 5% CO_2_ overnight. After the culture supernatants were aspirated, 100 μl of γδ T cell suspension (1.6 x 10^6^ cells/well) and 100 μl of a serially-diluted PTA, ZOL or HMBPP was added to each well in triplicate at concentrations of 0, 10^0^, 10^1^, 10^2^, 10^3^, 10^4^, 10^5^, 10^6^, or 10^7^ pM, or a serially-diluted anti-EGFR mAb at final concentrations of 0, 0.46, 1.37, 4.11, 12.3, 37, 111, 333 or 1000 ng/ml for HD06, HD07 and HDYT, or 78.125, 156.25, 312.5, 625, 1250, 2500, 5000 or 10000 ng/ml for HDYU. The plate was incubated at 37 °C with 5% CO_2_ for 48 h. Then, the culture supernatants were aspirated and the wells were gently washed three times with 200 μl of complete RPMI1640 medium. To the well was added 100 μl each of CellTiterGlo Reagent^®^ (PerkinElmer Inc.), and the cell lysates were transferred into a 96-well optiplate (PerkinElmer Inc.). Luminescence was measured through an ARVO or NIVO multi-plate reader (PerkinElmer Inc.).

### Enzyme-linked immunosorbent assay for IFN-γ

For measurement of IFN-γ production from γδ T cells in response to MPM cells in the absence or presence of PTA, ZOL, HMBPP or anti-EGFR mAb, γδ T cells (2 x 10^4^ cells in 100 μl of complete RPMI1640 medium) were placed in a 96-well flat-bottom plate (Corning Inc.), to which was added 100 μl each of PTA, ZOL or HMBPP at concentrations of 0, 0.1, 1 or 10 μM, or anti-EGFR mAb at concentrations of 0, 0.11, 0.33 or 1 μg/ml. The plate was incubated at 37°C with 5% CO_2_. After 48 h incubation, the cell suspensions were mixed and the plate was centrifuged at 600 x g at 4 °C for 2 min. The supernatants were transferred into a 96-well round bottom plate and placed at -80 °C overnight. The samples were thawed and interferon-γ (IFN-γ) levels were determined by enzyme-linked immunosorbent assay (ELISA, Peprotech, Rocky Hill, NJ) according to the manufacturer’s instructions.

### Statistical analysis

The statistical significance of differences was analyzed using GraphPad Prism ver. 9.0 with a *p* value less than 0.05 considered statistically significant.

## Results

### Expansion of peripheral blood γδ T cells derived from healthy donors

In order to examine effector functions of human γδ T cells against malignant pleural mesothelioma (MPM) cells, we first obtained peripheral blood from healthy adult volunteers and purified peripheral blood mononuclear cells (PBMC). The proportions of Vδ2^+^ T cells in lymphocyte fractions were 2.34%, 1.61%, 15.2%, and 13.9% for HD06, HD07, HD11, and HD12, respectively (**Supplementary Figure 1A**, upper panels). The majority of Vδ2^+^ T cells are known to express Vγ9 (also termed Vγ2) gene products and we hereafter use the term “γδ T cells” for Vγ9Vδ2-bearing T cells. When PBMC were stimulated with tetrakis-pivaloyloxymethyl 2-(thiazole-2-ylamino) ethylidene-1,1-bisphosphonate (PTA), a nitrogen-containing bisphosphonate prodrug, and expanded with IL-2 for 11 days, the proportions of γδ T cells were increased to 99.2%, 98.9%, 99.0% and 99.5% for HD06, HD07, HD11, and HD12, respectively (**Supplementary Figure 1A**, lower panels), consisting with previous studies. The numbers of γδ T cells before and after expansion were 1.07 x 10^6^ cells and 8.53 x 10^8^ cells for HD06 (797-fold expansion), 6.55 x 10^5^ cells and 1.50 x 10^9^ cells for HD07 (2290-fold expansion), 8.86 x 10^6^ cells and 3.73 x 10^9^ cells for HD11 (421-fold expansion), 2.05 x 10^6^ cells and 6.03 x 10^8^ cells for HD12 (294-fold expansion), respectively. It is worthy of note that a large number of highly purified γδ T cells could be obtained using the PTA/IL-2 stimulation/expansion system. Microscopic analysis revealed that cells started to form clusters in 3 to 5 days, depending on the initial proportion of γδ T cells in lymphocyte fractions (**Supplementary Figure 1B**).

On flow cytometric analysis, essentially all the expanded γδ T cells expressed NKG2D (CD314), an activating C-type lectin-like receptor and DNAX accessory molecule-1 (DNAM-1, CD226), a type I membrane protein containing 2 Ig-like C2-type domains as shown in **Supplementary Figure 1C** (upper and middle panels). In addition, γδ T cells expressed CD16 (also known as FcγRIII) to different degrees: weakly positive in HD06 and HD07 and highly positive in HD11 and HD12 (**Supplementary Figure 1C**, lower panels). In order to examine the effect of the types of anti-CD16 mAb on the expression pattern of CD16, HD11 and HD12 γδ T cells were stained with 6 different clones of anti-CD16 mAbs, resulting in essentially the same patterns of CD16 expression (**Supplementary Figure 1D**).

### Natural killer-like cellular cytotoxicity elicited by PTA-expanded γδ T cells derived from healthy donors

Since NKG2D, DNAM-1 and CD16 are known to be expressed on natural killer (NK) cells, we examined cytotoxic activity of the PTA/IL-2-expanded γδ T cells using a time-resolved fluorescence (TRF)-based assay system (12). It has been shown that γδ T cells exhibited potent cytotoxicity against Daudi Burkitt’s lympnoma cells and RPMI8226 multiple myeloma cells in a T cell receptor (TCR)-dependent manner (13). As shown in **Supplementary Figure 2A**, HD13 γδ T cells efficiently killed Daudi and RPMI8226 cells in an effector-to-target (E/T) ratio-dependent manner in 60 min, indicating that healthy donor-derived γδ T cells had capacity to kill tumor cells of hematopoietic origin in a short period of time.

It has been known that K562 erythrocytoma cells are susceptible to NK cells. We thus examined NK cell-like cytotoxic activity of healthy donor-derived γδ T cells against K562. As shown in **Supplementary Figure 2A**, K562 was killed by HD13 γδ T cells in an E/T ratio-dependent manner in a short period of time. The specific lysis reached to around 40% in 60 min at an E/T ratio of 40:1, equivalent to that to TCR-dependent cytotoxicity against Daudi cells. In addition, RAMOS-RAI, another Burkitt’s lymphoma cell line, was also efficiently killed by HD14 γδ T cells. We then examined susceptibility of U937 histocytoma cells to γδ T cells derived from various healthy donors. As shown in **Supplementary Figure 2B**, U937 was killed by HD13, HD14, HD15 and HD16 γδ T cells, while the cytotoxic effect of γδ T cells against U937 was somewhat weaker than that against tumor panels in **Supplementary Figure 1A**. Whereas it is, strictly speaking, difficult to distinguish between NK cell-like activity and TCR-dependent activity, it is evident that tumor cells of hematopoietic origin were sensitive to γδ T cells and the cytotoxic effect could be observed within 1 h at an E/T ratio of 40:1.

**Figure 1:**
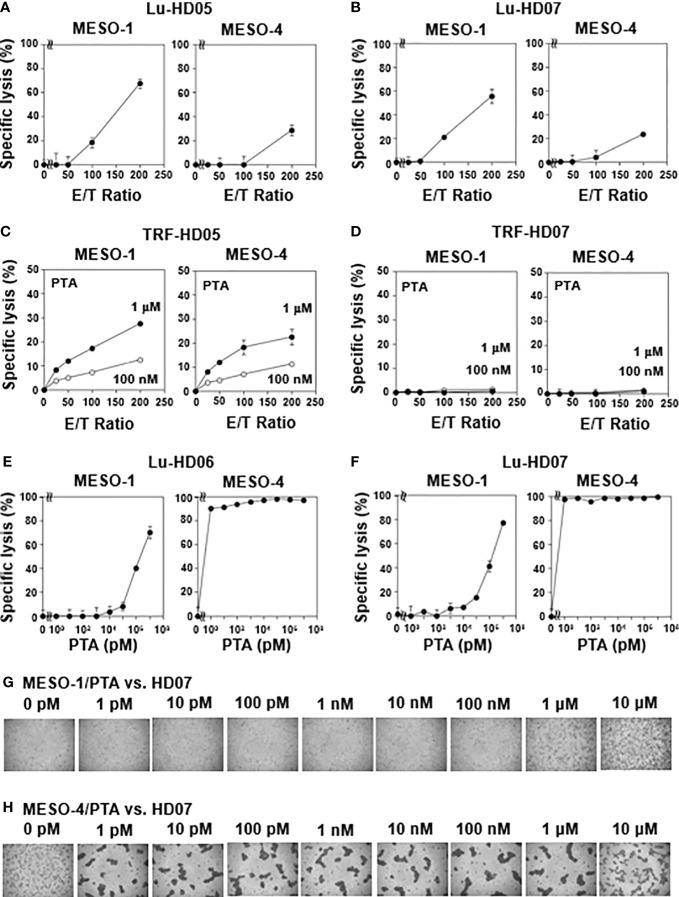
Natural killer-like activity and antigen-driven cytotoxicity against MPM cells exhibited by human γδ T cells. **(A, B)** Effect of effector-to-target ratios on cytotoxic activity of γδ T cells derived from healthy donors. MESO-1 and MESO-4 cells (2 x 10^4^ cells) were treated with a serial dilution of γδ T cells derived from healthy donors HD05 **(A)** or HD07 **(B)** at 37 °C for 72 h and specific lysis (%) was determined based on luciferase assay. **(C, D)** Effect of PTA on cytotoxicity elicited by γδ T cells derived from healthy donors. MPM cells (5 x 10^3^ cells) pulsed with PTA (100 nM or 1 μM) were challenged by γδ T cells derived from HD05 **(C)** or HD07 **(D)** at 37 °C for 1 h and specific lysis (%) was determined by Eu assay. **(E, F)** Effect of PTA on cytotoxic activity of γδ T cells derived from healthy donors. MPM cells (2 x 10^4^ cells) were incubated with γδ T cells derived from HD06 **(E)** or HD07 **(F)** in the presence of a serial dilution of PTA at 37 °C for 48 h and specific lysis (%) was determined by luciferase assay. **(G, H)** Microscopic analysis of MPM cells challenged by γδ T cells in the presence of PTA. Cell mixtures in **(F)** were observed under a microscope for MESO-1 **(G)** and MESO-4 **(H)**.

We next examined NK-like cytotoxic activity of γδ T cells against MESO-1 and MESO-4, malignant pleural mesothelioma cell lines using the same TRF-based assay system. When MPM cell lines were challenged by HD05 and HD07 γδ T cell lines for 1 h, detectable cytotoxicity was not observed even at an E/T ratio of 200:1 (**Supplementary Figure 2C**). As controls, MPM cells were challenged by NK cells derived from healthy donors, showing that NK cells exhibited cellular cytotoxicity against MPM cells to different degrees (**Supplementary Figure 2D**). We next set out to examine the effect of healthy donor-derived γδ T cells using a luminescence-based cytotoxic assay system that allowed us to determine cytotoxic activity of γδ T cells over a long period of time. When MPM cells were incubated with a serial dilution of HD05 (**Figure 1A**) or HD07 (**Figure 1B**) γδ T cells for 72 h, about 60% of MESO-1 cells and 30% of MESO-4 cells were killed at an E/T ratio of 200:1. It is worthy of note that a relatively high E/T ratio and a long duration of time are required for γδ T cells to kill MPM cells, when NK-like effector functions of γδ T cells were determined.

### TCR-dependent cytotoxicity against MPM cells by γδ T cells derived from healthy donors

We next examined TCR-dependent cytotoxic activity of healthy donor-derived γδ T cells against MPM cells. Since TCR-dependent cytotoxicity elicited by γδ T cells is expected to be more potent than NK-like activity, we again used TRF-based assay system to determine the specific lysis of PTA-pretreated MPM cells by γδ T cells in 1 h. When PTA is internalized into MPM cells, the prodrug is hydrolyzed by intracellular esterases to yield a biologically active N-BP, which increases intracellular concentrations of isopentenyl diphosphate (IPP) and dimethylallyl diphosphate (DMAPP) by inhibiting farnesyl diphosphate synthase (FDPS). The resulting IPP and DMAPP bind to the B30.2 domain of butyrophilin (BTN) 3A1. Then, γδ T cells are expected to recognize the PTA-sensitized MPM cells in a TCR- and BTN3A1/2A1-dependent manner. As shown in **Figure 1C**, a significant level of cytotoxicity was observed in an E/T ratio- and PTA concentration-dependent manner when PTA-sensitized MPM cells were challenged by HD05 γδ T cells under conditions used for TRF-based assay. In case of HD07 γδ T cells, cytotoxic activity against MESO-1 and MESO-4 was marginal under the same conditions as shown in **Figure 1D**.

We thus set out to examine TCR-dependent cytotoxicity of healthy donor-derived γδ T cells against MPM cells in a long period of time using the luminescence-based assay system. When MPM cells were incubated with HD06 γδ T cells at an E/T ratio of 80:1 in the presence of a serially diluted PTA for 48 h, MESO-1 cells were killed by HD06 γδ T cells in a PTA concentration-dependent manner, in which 1 μM or greater concentration of PTA was required for efficient killing. By contrast, PTA-sensitized MESO-4 cells were much more sensitive to HD06 γδ T cells, in which only 1 pM PTA was required for HD06 γδ T cells to kill MESO-4 cells (**Figure 1E**). Essentially the same results were obtained for HD07 γδ T cells as shown in **Figure 1F**. Microscopic analysis revealed that cell clustering of HD07 γδ T cells was observed when MESO-1 cells were pretreated with 1 μM or 10 μM (**Figure 1G**). In case of MESO-4 cells, as low as 1 pM PTA induced a significant level of cell clustering in HD07 γδ T cells, indicating that MESO-4 cells were more efficiently sensitized by PTA for γδ T cell-mediated cytotoxicity (**Figure 1H**).

We next examined TCR-dependent cytotoxicity of healthy donor-derived γδ T cells against ZOL-sensitized MPM cells. When MESO-1 cells were challenged by HD04 γδ T cells at an E/T ratio of 80:1 with a serially diluted ZOL for 48 h, a marginal level of cytotoxicity was noted at a ZOL concentration of 10 μM for MESO-1 (**Supplementary Figure 3A**, left). As for MESO-4 cells, a significant level of cytotoxicity was observed at 1 μM and 10 μM ZOL (**Supplementary Figure 3A**, right), indicating that the efficiency of MPM sensitization by ZOL was much lower than that by PTA (1 μM for ZOL vs. 1 pM for PTA). Essentially the same results were observed for HD07 γδ T cells (**Supplementary Figure 3B**). As shown in **Supplementary Figures 3C–F**, both HD04 and HD07 γδ T cells formed a cluster at ZOL concentrations where a significant level of specific lysis was induced **Supplementary Figures 3A, B**.

We also examined the effect of HMBPP in the same assay system, As shown in **Supplementary Figure 4A** (left), a moderate level of cytotoxicity was observed at an HMBPP concentration of 10 pM or greater, when MESO-1 cells were challenged by HD07 γδ T cells in the presence of a serially diluted HMBPP. In case of MESO-4, an HMBPP concentration of 10 nM or greater induced a significant level of cytotoxicity in HD07 γδ T cells (**Supplementary Figure 4A**, right). Similar results were observed for HD09 γδ T cells regarding cytotoxicity against HMBPP-sensitized MPM cells (**Supplementary Figure 4B**). Microscopic analysis demonstrated that HMBPP concentrations where a high level of specific lysis was induced triggered cell clustering in healthy donor-derived γδ T cells.

### CD16-dependent killing of MPM cells by γδ T cells derived from healthy donors

**Figure 2:**
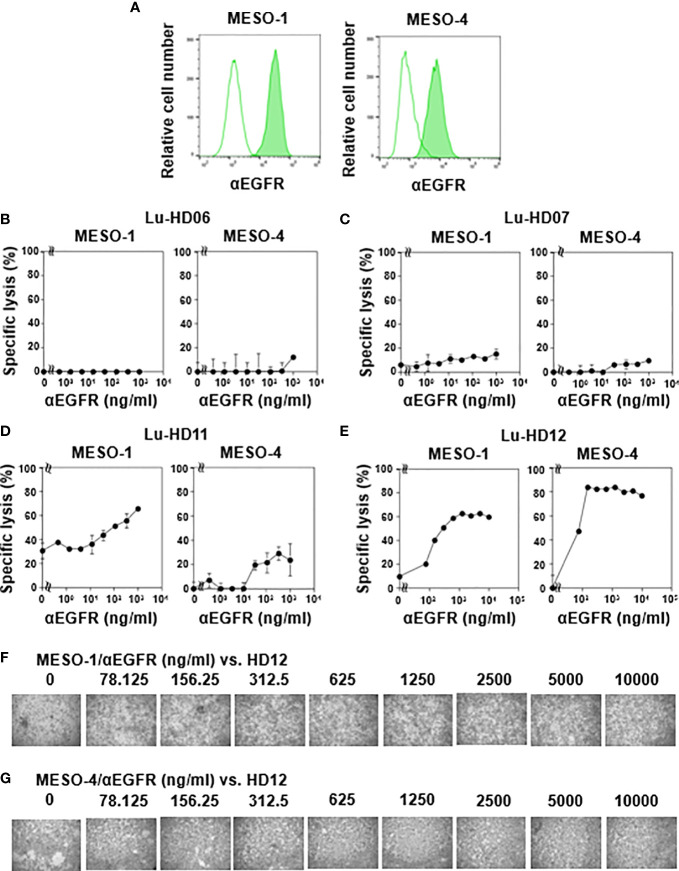
CD16-mediated cytotoxicity against MPM cells by γδ T cells. **(A)** Expression of EGFR on MPM cells. Flow cytometric analysis of MESO-1 and MESO-4 for the expression of EGFR. Open areas represent control staining and closed areas cell staining with anti-EGFR mAb. **(B–E)** Effect of expression levels of CD16 on cytotoxicity against MPM cells. MPM cells were challenged by CD16-low γδ T cells (HD06, B and HD07, C) or CD16-high γδ T cells (HD11, D and HD12, E) at 37 °C for 48 h and specific lysis (%) was determined based on luciferase assay. **(F, G)** Microscopic analysis of MPM cells challenged by γδ T cells in the presence of anti-EGFR mAb. Cell mixtures in **(E)** were observed under a microscope for MESO-1 **(F)** and MESO-4 **(G)**.

Since CD16 (also termed FcγRIII) expression was detected in γδ T cells derived from some healthy donors, we next analyzed antibody-dependent cellular cytotoxicity (ADCC) elicited by γδ T cells. Flow cytometric analysis revealed that both MESO-1 and MESO-4 expressed epidermal growth factor receptor (EGFR) (**Figure 2A**). Since HD06 and HD07 γδ T cells expressed only a marginal level of CD16, we first measured ADCC of these γδ T cell lines against MPM cells in the presence of serially diluted anti-EGFR mAb. As shown in **Figure 2B** (left panel), almost no cytotoxicity was observed when MESO-1 cells were incubated with HD06 γδ T cells in the presence of a serially diluted anti-EGFR mAb. As for MESO-4, only a marginal level of cytotoxicity was noted at mAb concentration of 1 μg/ml (**Figure 2B**, right panel). HD07 γδ T cells exhibited a low level of cytotoxicity against both MESO-1 and MESO-4 cells in the presence of a serially diluted mAb (**Figure 2C**). When HD11 and HD12 γδ T cells with relatively high levels of CD16 expression were examined for ADCC activity, a significant level of cytotoxicity was induced in these γδ T cells when incubated with MPM cells in the presence of a serially diluted mAb as shown in **Figures 2D, E**. It is worthy of note that γδ T cell clustering was apparently not observed under the conditions used in this study, suggesting that CD16-mediated killing of MPM cells is intrinsically different from TCR-mediated cytotoxicity against MPM cells (**Figures 2F, G**).

### IFN-γ production in response to MPM cells in γδ T cells derived from healthy donors

**Figure 3:**
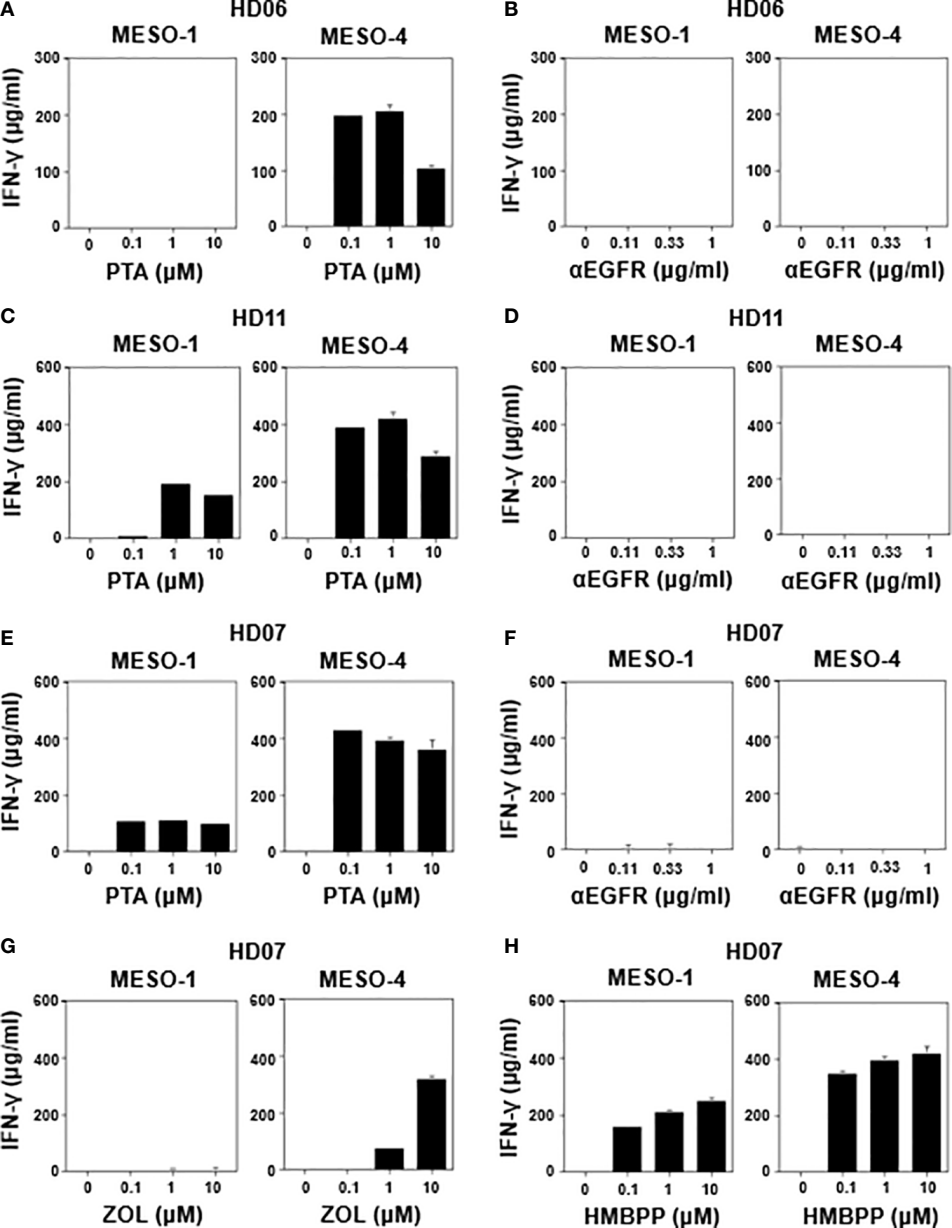
Effect of γδ T cell stimulants on the induction of IFN-γ secretion. MESO-1 or MESO-4 cells (2 x 10^4^ cells) were incubated with γδ T cells (1.6 x 10^6^ cells) derived from healthy donors (HD06 **(A, B)**, HD11 **(C, D)**, and HD07 **(E–H)**) in the presence of serial dilutions of stimulants (PTA **(A, C, E)**, ZOL **(G)**, HMBPP **(H)**, or anti-EGFR mAb **(B, D, F)**) at 37 °C for 48 h and the supernatants were examined for IFN-γ through ELISA.

We next determined IFN-γ production when MPM cells were challenged by healthy donor-derived γδ T cells in the presence of various stimulants. As shown in **Figure 3A** (right panel), HD06 γδ T cells produced a significant level of IFN-γ when incubated with MESO-4 cells in the presence of PTA at a concentration of 0.1 μM or greater, where the specific lysis was more than 90% (**Figure 1E**). By contrast, a detectable level of IFN-γ was not observed when MESO-1 cells were challenged by HD06 γδ T cells even in the presence of PTA at a concentration of 10 μM (**Figure 3A**, left panel), where specific lysis was around 70% (**Figure 1E**), suggesting that the production of IFN-γ was induced in HD06 γδ T cells, when the specific lysis (%) was significantly high.

We then examined IFN-γ production when MPM cells were incubated with HD06 γδ T cells in the presence of anti-EGFR mAb. As shown in **Figure 3B**, no detectable IFN-γ was observed even at an mAb concentration of 1 μg/ml, consisting with the results of specific lysis (**Figure 2B**), where essentially no ADCC activity was observed in HD06 γδ T cells with low CD16 expression. As shown in **Figure 3C**, HD11 γδ T cells secreted a significant level of IFN-γ when incubated with MESO-1 or MESO-4 cells in the presence of PTA. By contrast, essentially no IFN-γ production was observed when HD11 γδ T cells were incubated with MPM cells in the presence of anti-EGFR mAb (**Figure 3D**). Since HD11 γδ T cells expressed a high level of CD16 (**Supplementary Figure 1C**) and showed an ADCC activity (**Figure 2D**) against MPM cells in the presence of anti-EGFR mAb, it is most likely that γδ T cells do not secrete IFN-γ by stimulation through CD16.

When HD07 γδ T cells were incubated with MPM cells in the presence of PTA, a significant level of IFN-γ was observed (**Figure 3E**). By contrast, almost no IFN-γ secretion was observed when incubated with MPM cells in the presence anti-EGFR mAb (**Figure 3F**), which was consistent with the findings that HD07 γδ T cells expressed only a marginal level of CD16 (**Supplementary Figure 1C**) and exhibited only a marginal level of ADCC activity (**Figure 2C**) when incubated with MPM cells and anti-EGFR mAb. In addition, HD07 γδ T cells secreted IFN-γ when treated with MESO-4 cells and ZOL, whereas MESO-1 cells with ZOL failed to induce IFN-γ production in HD07 γδ T cells, probably because the inhibition of FDPS was not sufficient in MESO-1 cells to yield a significant level of IFN-γ production (**Figure 3G**). HMBPP induced IFN-γ production in HD07 γδ T cells in the presence of MPM cells (**Figure 3H**). Taken together, γδ T cells derived from healthy donors produced a significant level of IFN-γ when γδ T cells recognized phosphoantigen-pulsed MPM cells through γδ TCR and when the cellular cytotoxicity against MPM cells was relatively high, whereas CD16-dependent cellular cytotoxicity by γδ T cells against MPM cells was not correlated with IFN-γ production, suggesting that there was an intrinsic difference between effector functions mediated by γδ TCR and CD16 in γδ T cells.

### Expansion of γδ T cells derived from MPM patients

We next examined whether or not PTA induced expansion in γδ T cells derived from MPM patients. Peripheral blood samples were obtained from MPM patients, from which PBMC were purified and stimulated with PTA/IL-2 for 11 days. **Supplementary Figure 5** illustrates flow cytometric analysis of γδ T cells before and after expansion. The proportions of γδ T cells in lymphocyte fractions were 0.13%, 0.68%, 0.20%, and 0.11% for MPM01, MPM02, MPM03, and MPM04, respectively (**Supplementary Figure 5**, upper panels). When PBMC were stimulated with PTA/IL-2 for 11 days, the proportions of γδ T cells were increased to 15.7%, 83.3%, 71.7% and 20.6% for MPM01, MPM02, MPM03, and MPM04, respectively (**Supplementary Figure 5**, lower panels). The numbers of γδ T cells before and after expansion were 4.64 x 10^4^ cells and 3.30 x 10^7^ cells for MPM01 (710-fold expansion), 1.92 x 10^5^ cells and 1.75 x 10^8^ cells for MPM02 (911-fold expansion), 7.48 x 10^4^ cells and 4.73 x 10^7^ cells for MPM03 (632-fold expansion), 4.28 x 10^4^ cells and 1.98 x 10^7^ cells for MPM04 (463-fold expansion), respectively. It is worthy of note that PTA/IL-2 combination induced the expansion of MPM patient-derived γδ T cells to different degrees. Whereas the proportions of γδ T cells both before and after expansion were low, the expansion rates of MPM patients-derived γδ T cells were equivalent to those of healthy donor-derived γδ T cells.

### Effector functions of γδ T cells derived from MPM patients

**Figure 4:**
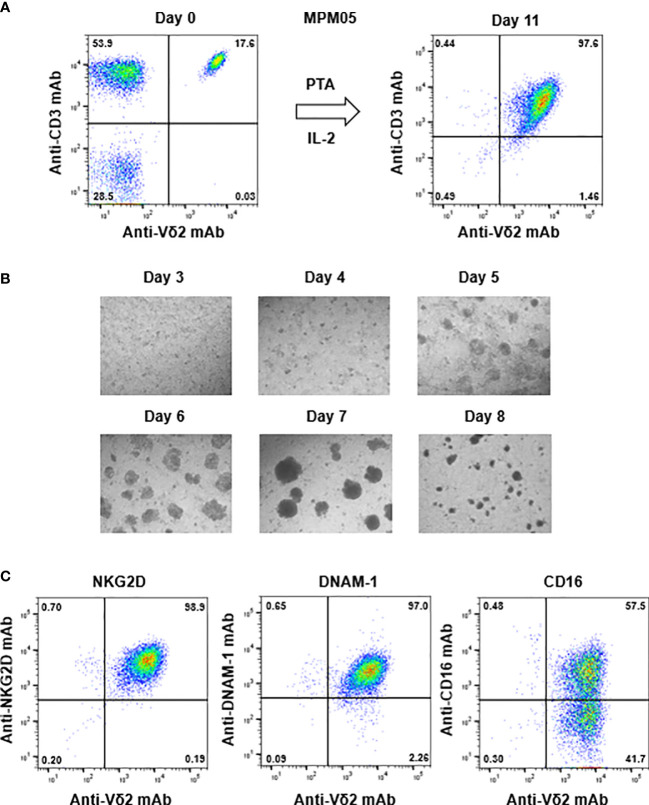
Expansion of γδ T cells from PBMC derived from an MPM patient. **(A)** PTA-driven expansion of γδ T cells. PBMC derived from MPM05, an MPM patient, was stimulated with PTA and expanded in the presence of IL-2 for 11 days. The purity of γδ T cells were monitored through flow cytometry. **(B)** Microscopic analysis of the time course of γδ T cells proliferation. MPM05-derived PBMC stimulated with PTA/IL-2 were monitored under a microscope on days 3, 4, 5, 6, 7 and 8. **(C)** Flow cytometric analysis of γδ T cells derived from MPM05. After expansion with PTA/IL-2 for 11 days, MPM05-derived γδ T cells were examined for the expression of NKG2D, DNAM-1 and CD16.

For example, the initial proportion of γδ T cells in MPM05 patients was 17.6% (**Figure 4A**, right panel), which was relatively higher than that (2.34%, 1.61%, 15.2%, and 13.9%) in healthy donors (**Supplementary Figure 1A**, upper panels) and the proportion after expansion with PTA/IL-2 was 97.6% (**Figure 4A**, left panel), which was equivalent to that (99.2%, 98.9%, 99.0%, and 95.5%) in healthy donors (**Supplementary Figure 1A**, lower panels). The expansion rate in MPM05 γδ T cells was 462-fold and cell clustering 5 to 8 days after stimulation was as vigorous as that of healthy donor-derived γδ T cells (**Figure 4B**), suggesting that proliferative responses of γδ T cells derived from MPM patients to phosphoantigens are intrinsically the same as those of healthy donor-derived γδ T cells. In addition, PTA/IL-2-expanded γδ T cells derived from MPM05 expressed a high level of NKG2D, DNAM-1 and CD16, suggesting that killing activity of MPM patient-derived γδ T cells might be equivalent to that of healthy donor-derived γδ T cells (**Figure 4C**).

**Figure 5:**
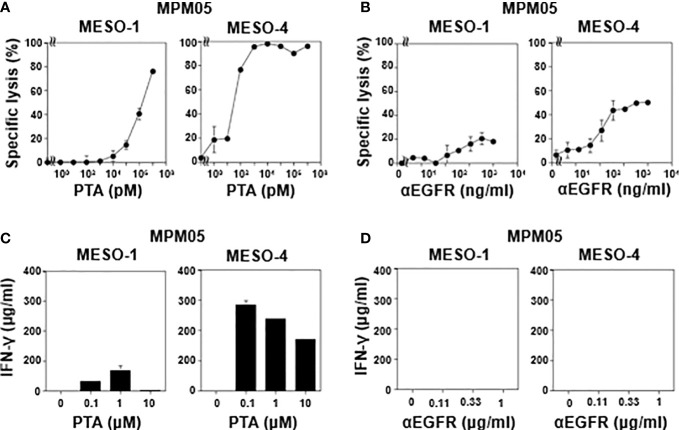
Effector functions of γδ T cells-derived from MPM05. **(A)** Effect of PTA on cytotoxic activity of MPM05-derived γδ T cells. MPM cells were challenged by γδ T cells derived from an MPM patient and specific lysis (%) were determined through luciferase assay. **(B)** Effect of anti-EFGR on cytotoxic activity of γδ T cells. MPM cells were incubated with γδ T cells derived from MPM05 and specific lysis (%) were determined through luciferase assay. **(C)** Effect of PTA on IFN-γ secretion from MPM05-derived γδ T cells. MPM cells were treated with γδ T cells derived from an MPM patient in the presence of a serial dilution of PTA and the supernatants were examined for IFN-γ. **(D)** Effect of anti-EFGR on the production of IFN-γ from γδ T cells. MPM cells were challenged by γδ T cells derived from MPM05 and the supernatants were examined for IFN-γ.

We then examined TCR-mediated cytotoxicity of MPM05-derived γδ T cells. As shown in **Figure 5A**, MPM05 γδ T cells exhibited a high level of specific lysis against both MESO-1 and MESO-4 cells in the presence of a serial dilution of PTA. MPM01- and MPM04-derived γδ T cells also exhibited cellular cytotoxicity against MESO-1 and MESO-4 cells, confirming that MPM patient-derived γδ T cells have potential to kill MPM cells. Furthermore, a significant level of ADCC activity was observed when both MESO-1 and MESO-4 cells were challenged by MPM05 γδ T cells in the presence of anti-EGFR mAb (**Figure 5B**). Regarding IFN-γ production, MPM05 γδ T cells secreted a significant level of IFN-γ in response to MESO-1 and MESO-4 in the presence of PTA (**Figure 5C**), whereas essentially no IFN-γ was produced when MPM cells in the presence of anti-EGFR mAb were challenged by MPM05 γδ T cells (**Figure 5D**), consistent with the findings in healthy donor-derived γδ T cells, strongly suggesting that both healthy donor-derived and MPM patient-derived γδ T cells would be utilized for adoptive immunotherapy for MPM.

## Discussion

Malignant pleural mesothelioma (MPM) is an intractable malignancy and characterized by its heterogeneity among patients and even within individual tumors (14). Conventional treatment for MPM is a combination therapy of cisplatin plus pemetrexed (3–5). In 2020, a combination of anti-PD-1 mAb nivolumab plus anti-CTLA-4 mAb ipilimumab was approved by the U.S. Food and Drug Administration (FDA) as a first-line therapy for unresectable MPM (7, 15, 16). Whereas initial promising results of mAb-based therapy have changed the landscape of MPM treatment, the efficacy is limited to a certain subset of patients, indicating an urgent need to establish biomarkers for patient selection (17–24).

Recent advances in gene sequence technology and transcriptome analyses revealed that MPM cells have mutations in tumor-suppressor genes, such as BAP1, NF2, RASSF1, LATS2WT1, p16, TP53, and CDKN2A, whereas it is not clear molecular targeting strategy is effective for the eradication of MPM cells (25–27). The variation in MPM with different treatment-responses suggests the need for the development of immunotherapy, especially adoptive cell therapy, because small-molecule drugs targeting specific molecules in certain populations of tumor cells might fail to exhibit antitumor effects on other variant tumors.

Recently, much attention has been paid to cell-based therapies for MPM (28, 29). A variety of cell-based therapies have been developed in the last decade, among which chimeric antigen receptor (CAR) T cells are the most successful measures for the treatment of B lymphoma. Although CAR T cell therapy is efficacious for B lymphoma, solid tumors are generally resistant to this treatment, intriguing us to develop novel cell-based therapies. γδ T cells have attracted considerable attention as a unique immune effector cell population (30–32). αβ T cells recognize antigenic peptide in the context of MHC class I or II molecules with the help of CD8 or CD4 coreceptors. By contrast, most γδ T cells are CD4^-^CD8^-^ and not restricted by MHC. In addition, the majority of γδ T cells expressing Vγ9 (also termed Vγ2) and Vδ2 gene products recognize so-called phosphoantigens, such as HMBPP as a foreign antigen and IPP and DMAPP as self antigens (13, 33–37). Recent studies demonstrated that BTN3A1 and BTN2A1 play an essential role in the recognition of phosphoantigens by γδ T cells (38–45).

In the present study, we first demonstrated that healthy donor-derived γδ T cells were efficiently expanded using our PTA/IL-2 expansion system, resulting in a large number of highly purified γδ T cells (ca. 1000-fold expansion with purities up to > 95%) without any purification steps in 11 days, consistent with previous reports (46). Since PTA/IL-2-expanded γδ T cells expressed a high level of NK receptors such as NKG2D and DNAM-1, we examined the cytotoxic activity of γδ T cells against various tumor cell lines. γδ T cells exhibited a significant level of cytotoxic activity against tumor cells of hematopoietic origin within 1 h at an E/T ratio of 40:1. Since phosphoantigens were not included in this assay system, this activity is considered to be NK-like cytotoxicity. However, γδ T cells failed to kill MPM cells under the same conditions. Even if the E/T ratio was set at 200:1, essentially no cytotoxicity was observed, suggesting that MPM cells were resistant to γδ T cell-mediated NK-like cytotoxicity in a short period of time and at conventional E/T ratios.

We thus employed an alternative luminescence-based assay system that allowed us to determine cell-mediated cytotoxicity over several days. In this study, we employed a time-resolved fluorescence-based assay using a non-radioactive Eu-chelate labeling system for the short period assay, which could be used for the cellular cytotoxicity assay for 20 min to 2 h, because the spontaneous release of the chelate compound is relatively fast. By contrast, the luminescence-based assay using a luciferase could be used for the long period assay for 4 h to 3 days. When the incubation time was extended to as long as 72 h and the E/T ratio was increased up to 200:1, moderate levels of NK-like cytotoxicity of γδ T cells against MPM cells were observed, indicating that γδ T cells required relatively a long duration of time (up to 72 h) and a high E/T ratio (up to 200:1) to kill MPM cells in an NK cell-like manner, compared to those for the killing of tumor cells of hematopoietic origin. It is thus essential to artificially modulate the efficacy of γδ T cell-mediated killing for the development of adoptive transfer therapy as a measure to treat MPM, because a large number of γδ T cells and long-time contact between γδ T cells and tumor cells are required for tumor cell lysis by γδ T cells.

Since γδ T cells exert potent cytotoxicity against tumor cells when TCR-mediated signals are transduced, we used three representative stimulators for γδ T cells, PTA (an N-BP prodrug), HMBPP (a pyrophosphomonoester antigen) and ZOL (an N-BP drug). It is worthy of note that γδ T cells killed 20 – 30% of MPM cells in the presence of 1 μM of PTA at an E/T ratio of 200:1 within 1 h. When the incubation time was extended to 48 h, more than 90% of MPM cells were killed in the presence of as low as 1 pM of PTA, suggesting that adoptive transfer of γδ T cells together with a low concentration of PTA might lead to the eradication of MPM cells *in vitro*.

We further examined the effect of HMBPP and ZOL, since several clinical trials have been carried out using phosphoantigens and N-BPs (47). Whereas HMBPP and ZOL are ideal antigens to stimulate peripheral blood γδ T cells, MPM cells are not efficiently sensitized by them, possibly because fluid-phase endocytosis in MPM cells is not efficient, compared to that in macrophages and dendritic cells. Especially, ZOL seemed not to be efficiently engulfed by MPM cells, resulting in poor specific lysis when MPM cells were challenged by γδ T cells in the presence of ZOL.

We then examined ADCC elicited by γδ T cells against MPM cells, since γδ T cells derived from some donors expressed CD16 (FcγIII) and a previous study indicated that anti-EGFR mAb is effective for treatment of MPM in a mouse model (48). When MPM cells were challenged by CD16-high γδ T cells in the presence of anti-EGFR mAb, a significant level of ADCC was observed. By contrast, CD16-low γδ T cells failed to show ADCC against MPM cells. It is interesting that γδ T cells did not form clusters during ADCC responses. In addition, IFN-γ production was also not detected even if a significant level of cytotoxicity was observed. This is in contrast to PTA-driven TCR-mediated killing of MPM cells by γδ T cells, in which large γδ T cell clusters were formed and a significant amount of IFN-γ was produced by γδ T cells. When the clinical application is considered, anti-EGFR mAb administration together with γδ T cells might be milder and safer than TCR stimulators plus γδ T cell therapy, since mAb/CD16-mediated therapy might not induce inflammatory cytokines.

We next examined the expansion efficiency and killer activity of MPM patient-derived γδ T cells. Five MPM patients were recruited to this study, four of which exhibited relatively low initial proportions of γδ T cells. When stimulated with PTA/IL-2 for 11 days, the purities of γδ T cells vary in the five individuals, but the expansion rates were equivalent to those of healthy donor-derived γδ T cells, suggesting that MPM patient-derived γδ T cells were immunologically functional, even though the initial proportions were low. MPM5 patient exhibited a high initial proportion of γδ T cells and the expansion patterns and effector functions of MPM5 γδ T cells were equivalent to those of healthy donors. These findings suggest that both autologous and allogeneic γδ T cells might be used for adoptive cell therapy for MPM. When initial proportions of γδ T cells are high, it is reasonable to use autologous γδ T cells for adoptive cell therapy. In case of patients with low initial proportions of γδ T cells, both autologous and allogeneic γδ T cells could be used for cell therapy, because MHC molecules are not involved in the effector functions of γδ T cells. Taken together, γδ T cell-based therapy is a promising measure for cell-based MPM therapy and combined therapies with anti-EGFR mAb or TCR signaling mediators might improve the efficacy of γδ T cell-based therapy.

## Data availability statement

The raw data supporting the conclusions of this article will be made available by the authors, without undue reservation.

## Ethics statement

The studies involving human participants were reviewed and approved by Institutional Review Board of Nagasaki University Hospital. The patients/participants provided their written informed consent to participate in this study.

## Author contributions

YT, HTa, ST and HM designed the research. YU, HG, HS, HTo, MT, MI, and YT performed the experiments. YU, HY, YT and HM prepared the manuscript. YT, HTa and HM supervised the overall project. All authors contributed to the article and approved the submitted version.
