# Supplementary material for: Three distinct mechanisms underlying human γδ T cell-mediated cytotoxicity against malignant pleural mesothelioma
Source: Front Immunol. 2023 Mar 17;14:1058838. doi: 10.3389/fimmu.2023.1058838 (PMC10063812; doi:10.3389/fimmu.2023.1058838)
Supplement: Supplementary file 1 [file DataSheet_1.pdf]

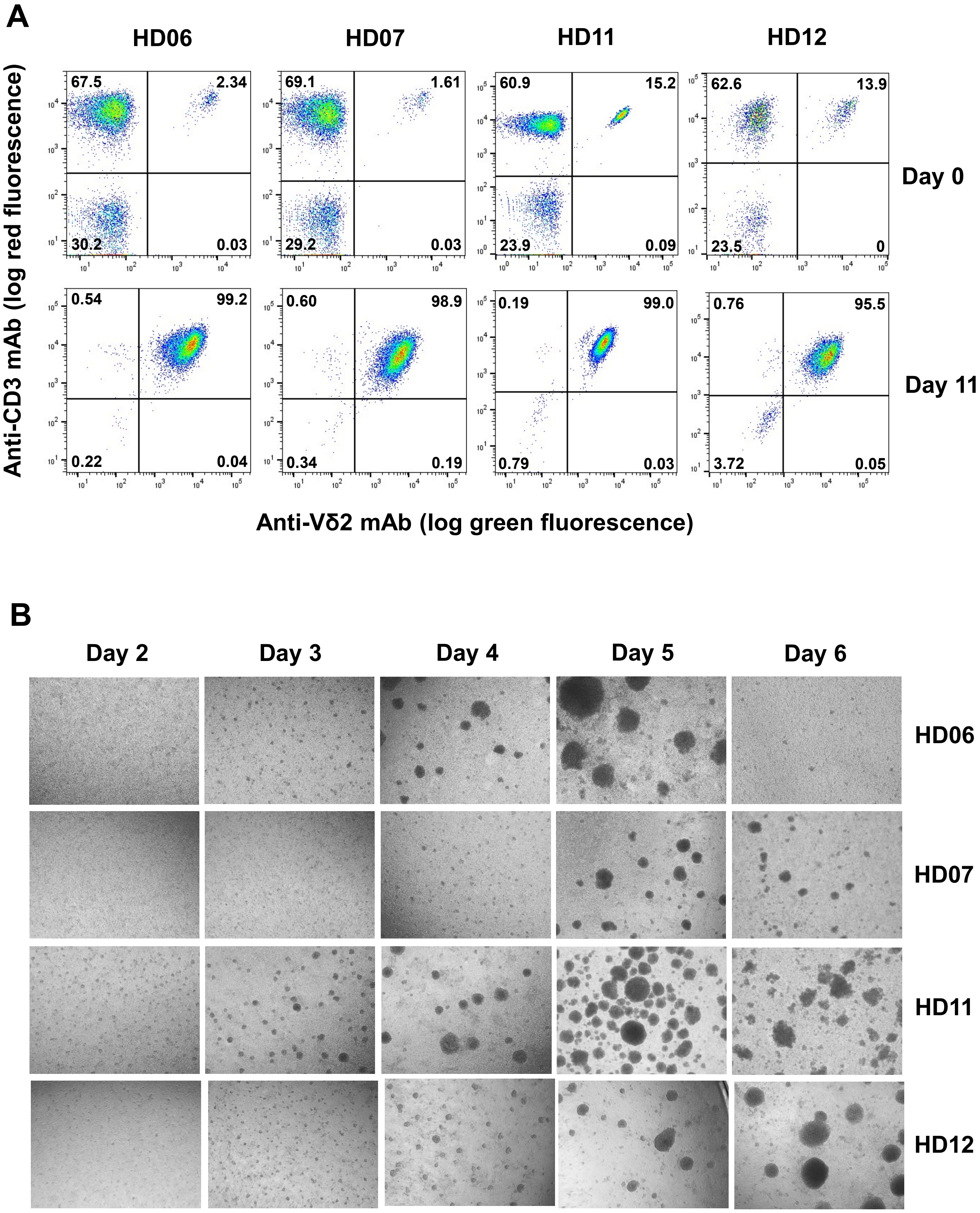

A
HD06
HD07
HD11
HD12
67.5
2.34
30.2
0.03
69.1
1.61
29.2
0.03
60.9
15.2
23.9
0.09
62.6
13.9
23.5
0
Day 0
0.54
99.2
0.22
0.04
0.60
98.9
0.34
0.19
0.19
99.0
0.79
0.03
0.76
95.5
3.72
0.05
Day 11
Anti-CD3 mAb (log red fluorescence)
Anti-Vδ2 mAb (log green fluorescence)
B
Day 2
Day 3
Day 4
Day 5
Day 6
HD06
HD07
HD11
HD12

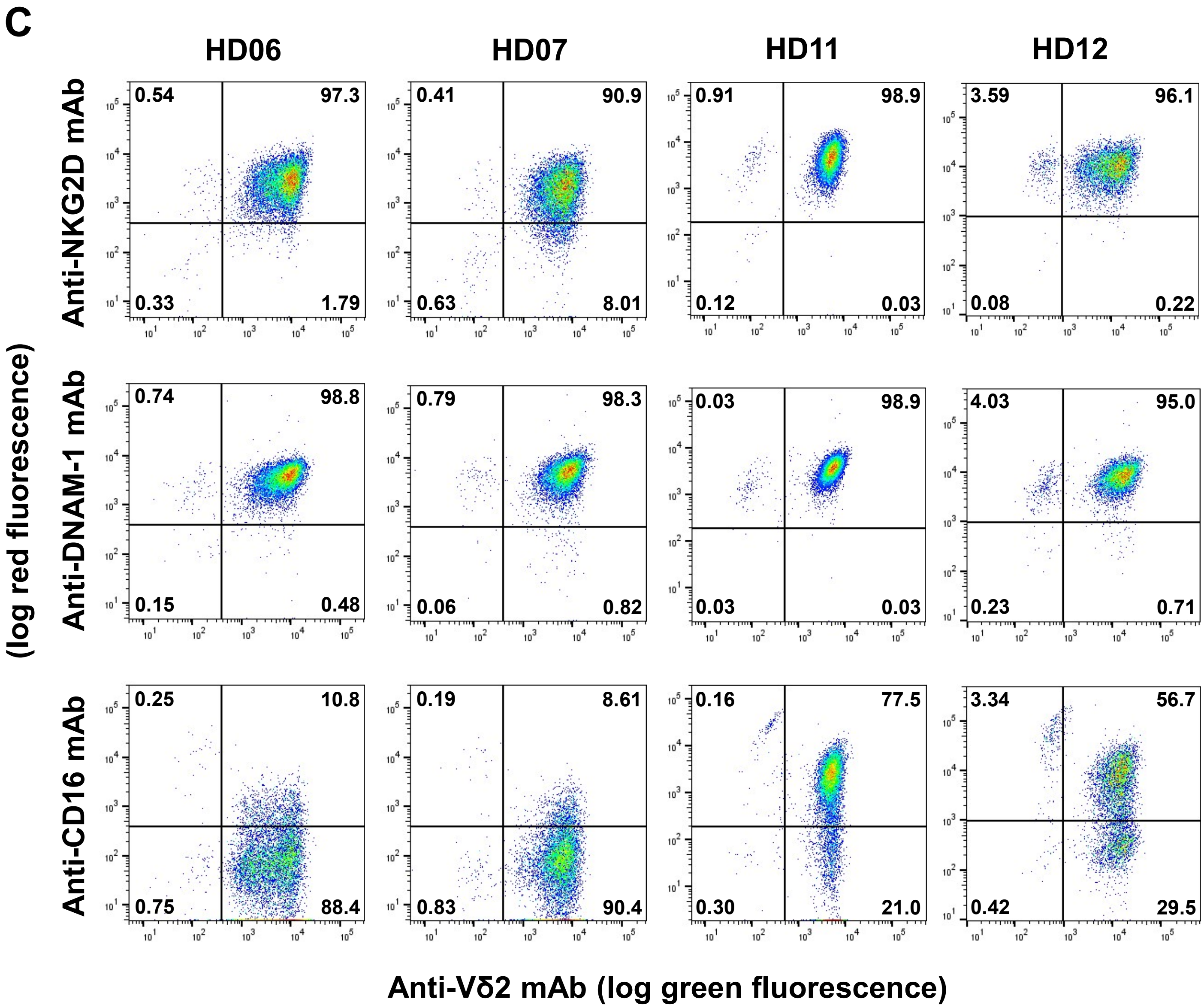

C
HD06
HD07
HD11
HD12
Anti-NKG2D mAb
Anti-DNAM-1 mAb
Anti-CD16 mAb
(log red fluorescence)
0.54
97.3
0.33
1.79
0.41
90.9
0.63
8.01
0.91
98.9
0.12
0.03
3.59
96.1
0.08
0.22
0.74
98.8
0.15
0.48
0.79
98.3
0.06
0.82
0.03
98.9
0.03
0.03
4.03
95.0
0.23
0.71
0.25
10.8
0.75
88.4
0.19
8.61
0.83
90.4
0.16
77.5
0.30
21.0
3.34
56.7
0.42
29.5
Anti-Vδ2 mAb (log green fluorescence)

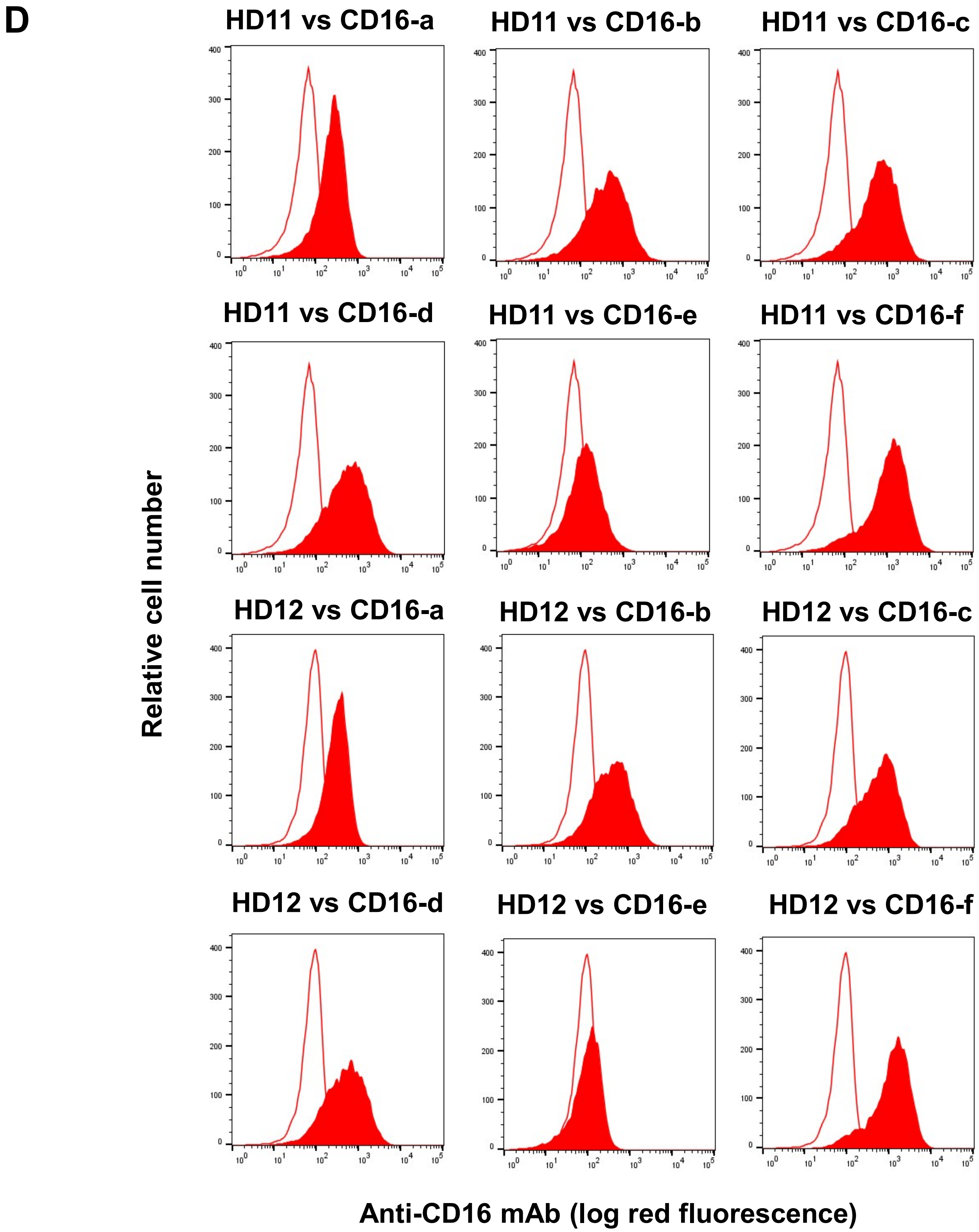
D
HD11 vs CD16-a
HD11 vs CD16-b
HD11 vs CD16-c
HD11 vs CD16-d
HD11 vs CD16-e
HD11 vs CD16-f
HD12 vs CD16-a
HD12 vs CD16-b
HD12 vs CD16-c
HD12 vs CD16-d
HD12 vs CD16-e
HD12 vs CD16-f
Relative cell number
Anti-CD16 mAb (log red fluorescence)

Supplementary Fig. 1. Expansion of γδ T cells by PTA/IL-2. (A) Flow cytometric analysis of PTA-expanded γδ T cells derived from healthy donors. Peripheral blood samples (10 ml) were collected from four healthy donors (HD06, 07, 11 and 12), to which was added 10 ml each of PBS. The diluted blood (20 ml) was loaded onto 20 ml of Ficoll-Paque™ PLUS (Cytiva, Shinjuku-ku, Tokyo, Japan) in a 50 ml conical tube (Corning Inc., Corning, NY), which was centrifuged at 600 x g and 20 °C for 30 min. After being washed two times with PBS, the cells were resuspended in Yssel's medium supplemented with 10% human AB serum (Yssel, H., *et al.* Serum-free medium for generation and propagation of functional human cytotoxic and helper T cell clones. ***J. Immunol. Methods*** 72:219-227, 1984). Before and after expansion with PTA/IL-2, the cells were stained with phycoerythrin (PE)-labeled anti-CD3 mAb and fluorescein isothiocyanate (FITC)-labeled anti-Vδ2 mAb and analyzed through a FACS Lyric flow cytometer (BD Biosciences, Franklin Lakes, NJ). **(B) PTA-mediated clustering of γδ T cells.** After stimulation with PTA/IL-2, the cell clustering was monitored under a microscope equipped with a CCD camera. **(C) Flow cytometric analysis of cell surface markers on PTA-expanded γδ T cells.** After expansion with PTA/IL-2, the cells were stained with phycoerythrin (PE)-labeled anti-NKG2D, DNAM-1, or CD16 mAb and fluorescein isothiocyanate (FITC)-labeled anti-Vδ2 mAb and analyzed through a FACS Lyric flow cytometer. **(D) Flow cytometric analysis of the expression of CD16 on PTA-expanded γδ T cells.** After expansion with PTA/IL-2, the cells derived from HD11 and HD12 were stained with phycoerythrin (PE)-labeled CD16 mAb derived from various hybridoma clones and fluorescein isothiocyanate (FITC)-labeled anti-Vδ2 mAb and Vδ2-bearing γδ T cells were analyzed for the expression of CD16 through a FACS Lyric flow cytometer. (a) PE-labeled anti-human CD16 mAb clone: EPR22409-124, isotype: recombinant rabbit mAb, Abcam, Cambridge, UK, (b) clone: 3G8, isotype: mouse IgG1, κ, BD Biosciences, Franklin Lakes, NJ, (c) clone: eBioCB16, isotype: mouse IgG1, κ, ThermoFisher Scientific, Waltham, MA, (d) clone: REA423, recombinant human IgG1, Miltenyi Biotec B.V. & Co., KG, Bergisch Gladbach, Germany, (e) clone: B73.1, isotype: mouse IgG1, κ, BioLegend, San Diego, CA, or (f) clone: 3G8, isotype: mouse IgG1, κ, BioLegend, San Diego, CA.

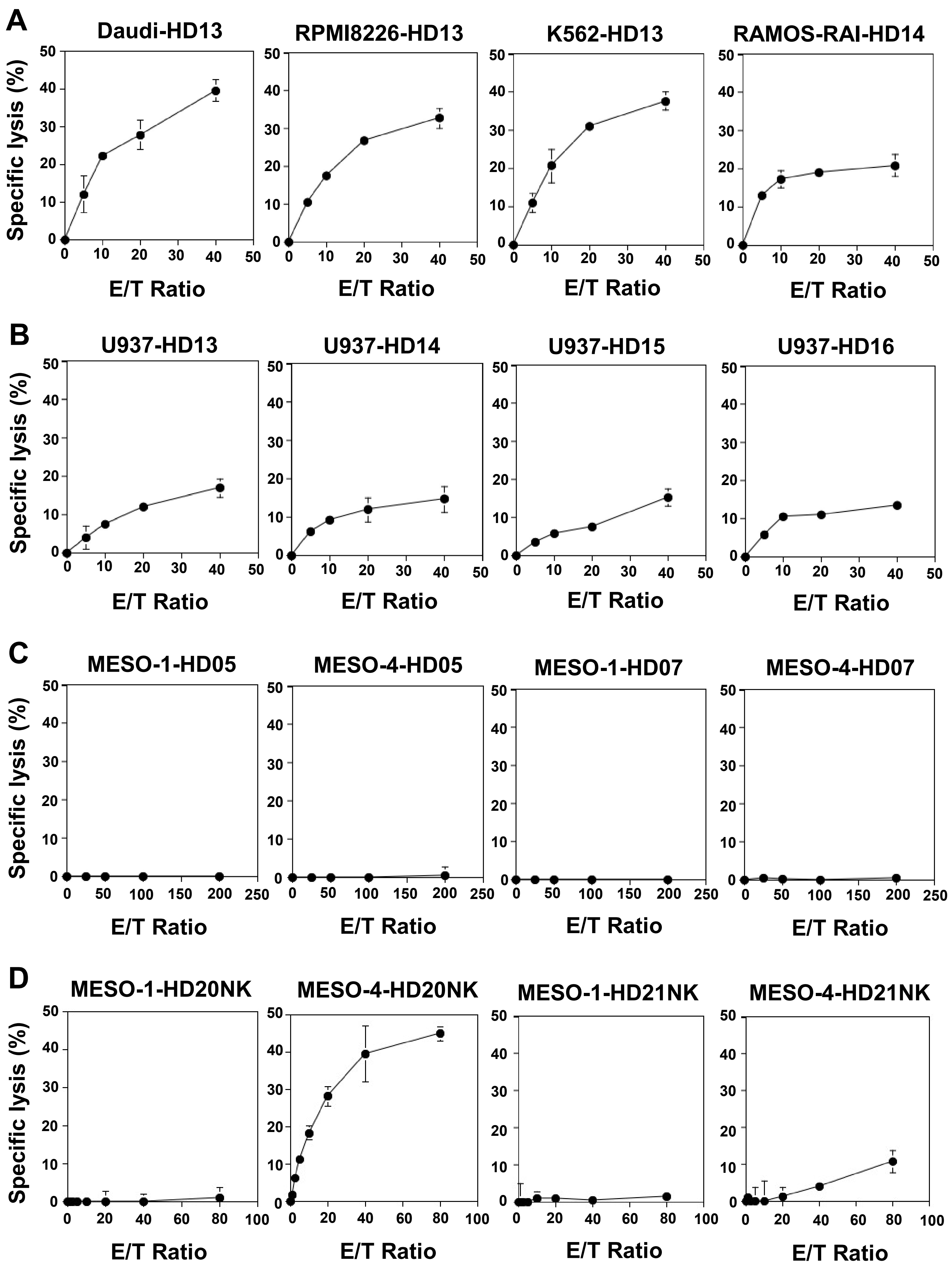
A
Daudi-HD13
RPMI8226-HD13
K562-HD13
RAMOS-RAI-HD14
B
U937-HD13
U937-HD14
U937-HD15
U937-HD16
C
MESO-1-HD05
MESO-4-HD05
MESO-1-HD07
MESO-4-HD07
D
MESO-1-HD20NK
MESO-4-HD20NK
MESO-1-HD21NK
MESO-4-HD21NK
Specific lysis (%)
E/T Ratio

Supplementary Fig. 2. Comparison of γδ T cell-mediated cytotoxicity against tumor cells of hematopoietic origin and MPM cells. (A) Cytotoxicity exhibited by γδ T cells derived from healthy donors against hematopoietic tumor cells. Daudi, a Burkitt's lymphoma cell line, RPMI8226, a multiple myeloma cell line, K562, an erythrocytoma cell line, and RAMOS-RAI, a Burkitt's lymphoma cell line were challenged by PTA-expanded γδ T cells at effector-to-target (E/T) ratios of 40:1, 20:1, 10:1, and 5:1 for 40 min at 37°C with 5% CO_2 and specific lysis (%) was determined using a europium (Eu)-chelate complex-based time-resolved fluorescence assay system. **(B) Cytotoxic activity against U937 cells by PTA-expanded γδ T cells.** U937, a diffuse histocytic lymphoma cell line, was treated with healthy donor-derived γδ T cells and specific lysis (%) was determined as described in (A). **(C) Effect of γδ T cells on MPM cells.** Two MPM cell lines, MESO-1 and MESO-4, were incubated with γδ T cells derived from healthy donors and specific lysis (%) was determined at effector-to-target (E/T) ratios of 200:1, 100:1, 50:1, and 25:1. **(D) Effect of NK cells on MPM cells.** Two MPM cell lines, MESO-1 and MESO-4, were incubated with NK cells derived from healthy donors and specific lysis (%) was determined at effector-to-target (E/T) ratios of 80:1, 40:1, 20:1, 10:1, 5:1, 2.5:1, and 1.25:1.

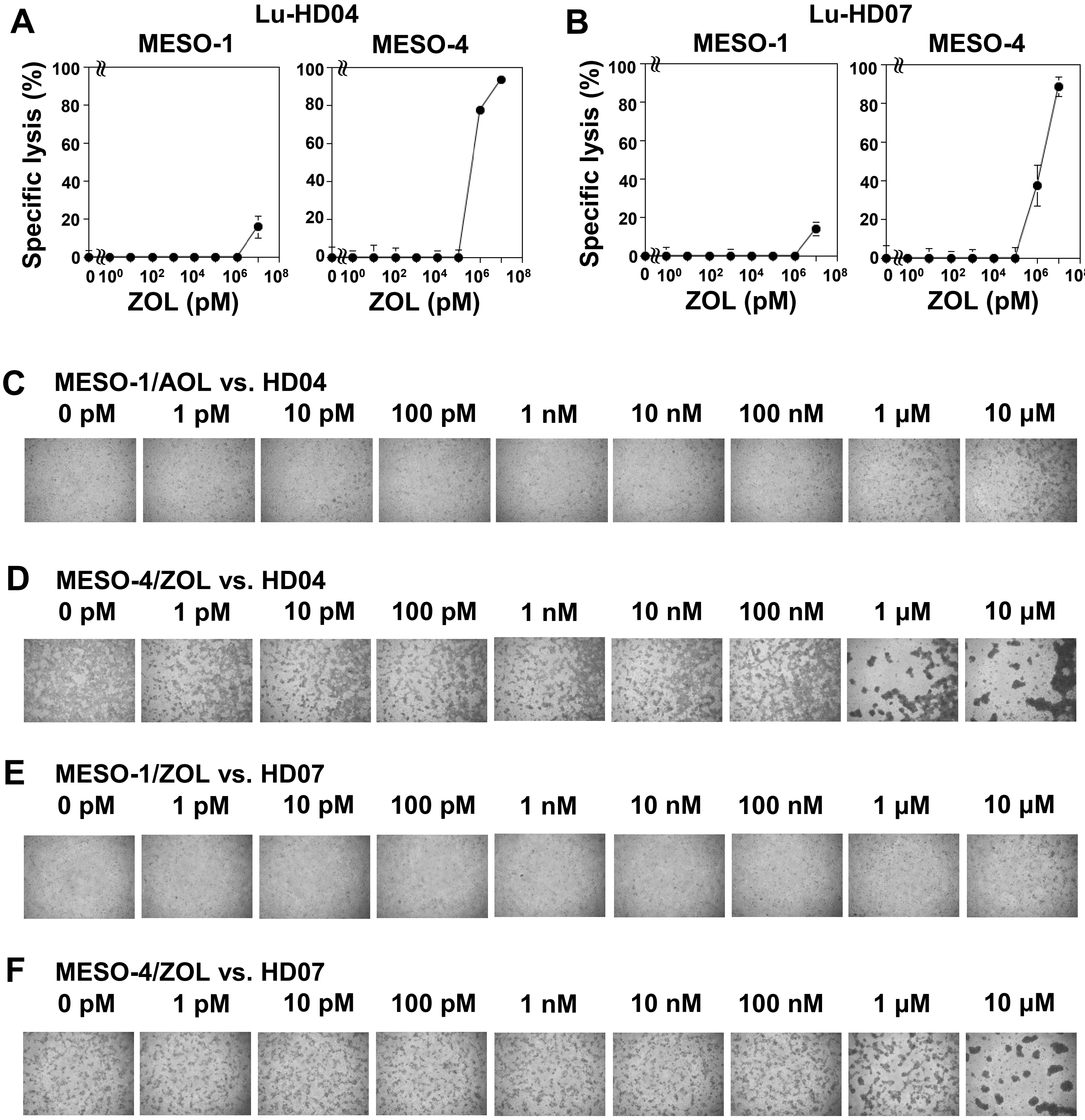

Supplementary Fig. 3. Effect of ZOL on cytotoxicity against mesothelioma elicited by γδ T cells derived from healthy donors. MESO-1 and MESO-4 (2×10^4 cells) were challenged by $V\delta2^+$ T cells (1.5×10^6 cells) derived from HD04, a healthy donor, in the presence of a serial dilution of ZOL for 48 h and cellular cytotoxicity was determined by luciferase-based assay (A). The same assay was performed for with HD07, another healthy donor (B). The cell suspensions after 48 in (A) and (B) were photographed under an Olympus microscope equipped with a CCD camera: (C) MESO-1 vs. HD04, (D) MESO-4 vs. HD04, (E) MESO-1 vs. HD07, and (F) MESO-4 vs. HD07.,

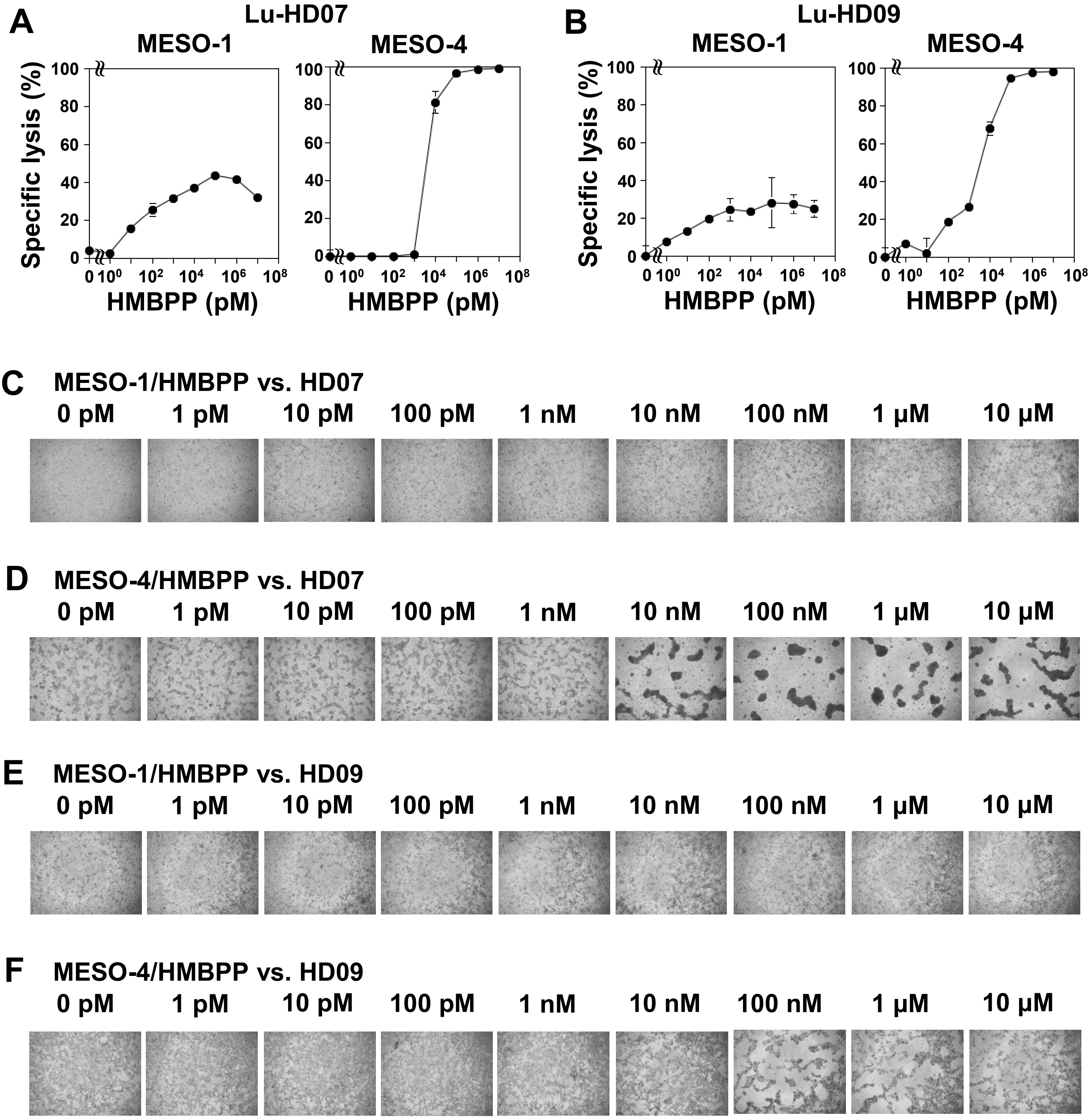

Supplementary Fig. 4. Effect of HMBPP on cytotoxicity against mesothelioma elicited by γδ T cells derived from healthy donors. MESO-1 and MESO-4 (2×10^4 cells) were challenged by $V\delta2^+$ T cells (1.5×10^6 cells) derived from HD07, a healthy donor, in the presence of a serial dilution of HMBPP for 48 h and cellular cytotoxicity was determined by luciferase-based assay (A). The same assay was performed for with HD09, another healthy donor (B). The cell suspensions after 48 in (A) and (B) were photographed under an Olympus microscope equipped with a CCD camera: (C) MESO-1 vs. HD07, (D) MESO-4 vs. HD07, (E) MESO-1 vs. HD09, and (F) MESO-4 vs. HD09.

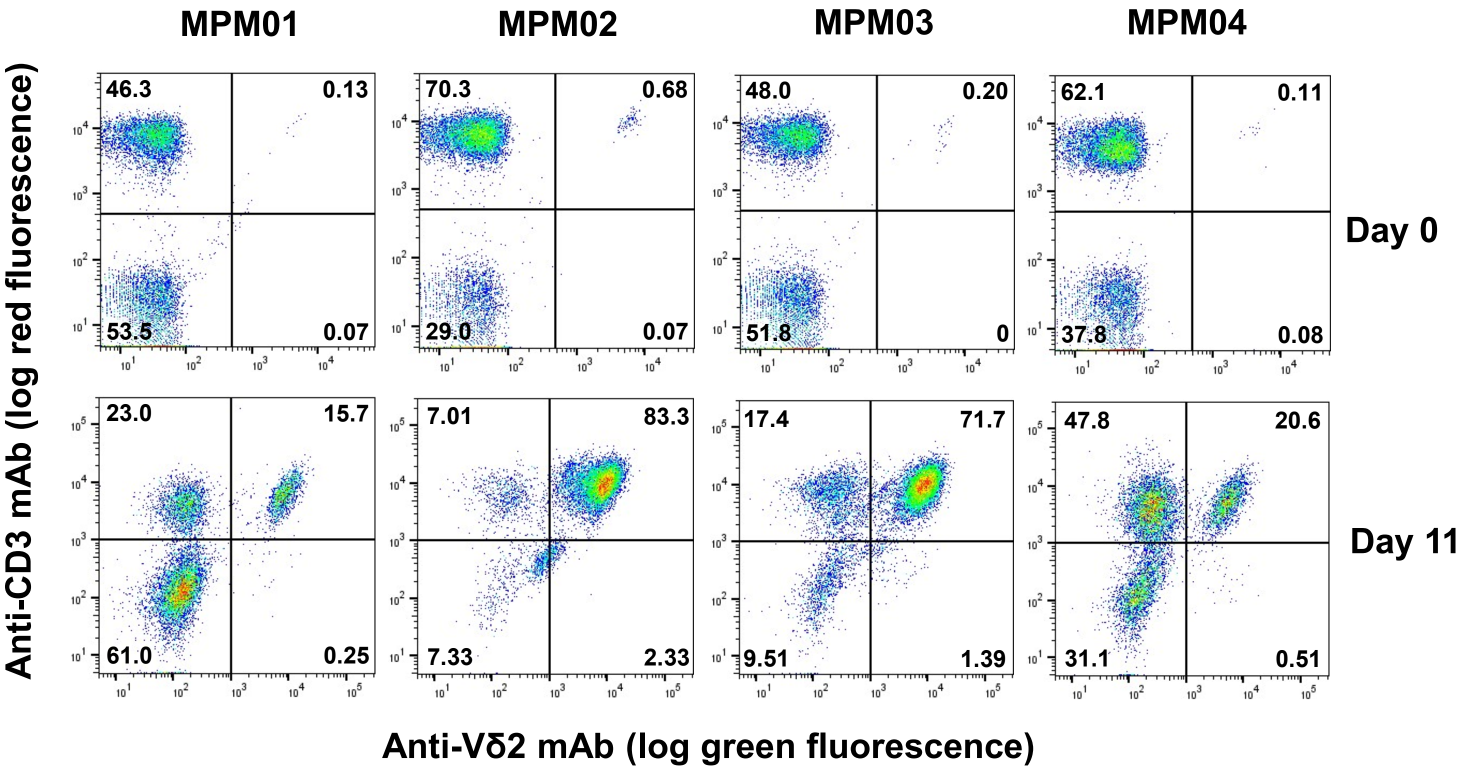

Supplementary Fig. 5. Flow cytometric analysis of PTA-expanded γδ T cells derived from patients with malignant pleural mesothelioma. Peripheral blood samples (10 ml) were collected from four patients with malignant pleural mesothelioma (MPM01-MPM04), to which was added 10 ml each of PBS. The diluted blood (20 ml) was loaded onto 20 ml of Ficoll-Paque^{TM} PLUS in a 50 ml conical tube, which was centrifuged at 600 x g and 20°C for 30 min. After being washed two times with PBS, the cells were resuspended in 7.2 ml of Yssel's medium supplemented with 10% human AB serum. Before and after expansion with PTA/IL-2, the cells were stained with phycoerythrin (PE)-labeled anti-CD3 mAb and fluorescein isothiocyanate (FITC)-labeled anti-Vδ2 mAb and analyzed through a FACS Lyric flow cytometer.

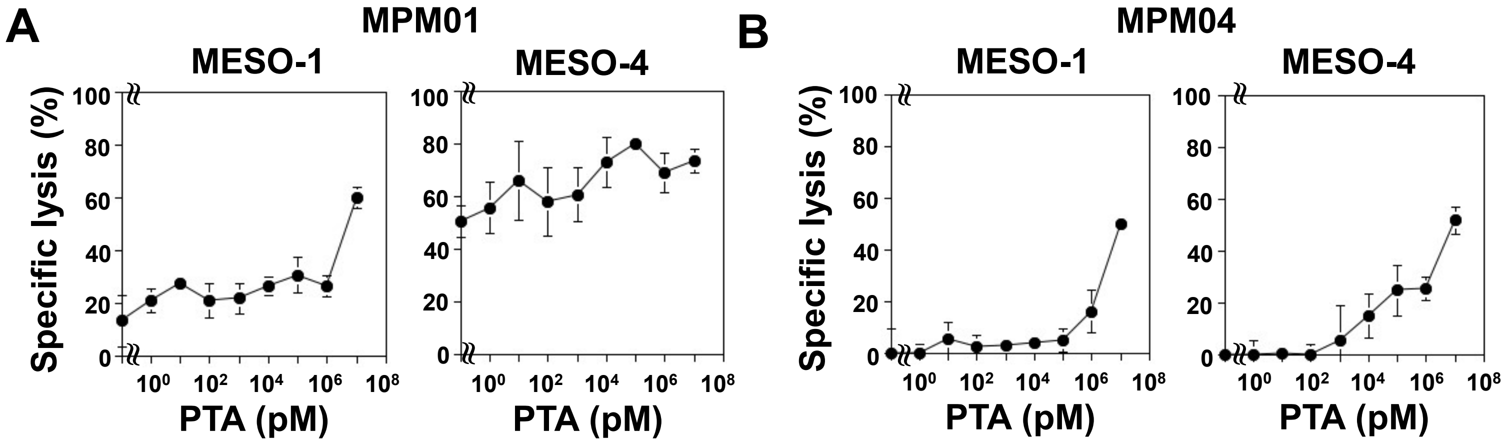

Supplementary Fig. 6. Effect of PTA on cytotoxicity against mesothelioma elicited by γδ T cells derived from MPM patients. MESO-1 and MESO-4 (2 x 10^4 cells) were challenged by $V\delta2^+$ T cells (1.5 x 10^6 cells) derived from an MPM patient, MPM01 in the presence of a serial dilution of PTA for 48 h and cellular cytotoxicity was determined by luciferase-based assay (A). The same assay was performed for with MPM04, another MPM patient (B).
